# The ‘president’s drug’

**DOI:** 10.1007/s12471-020-01441-x

**Published:** 2020-07-10

**Authors:** A. A. M. Wilde, J. A. Offerhaus

**Affiliations:** Department of Clinical and Experimental Cardiology, Amsterdam UMC, Heart Centre, Amsterdam Cardiovascular Sciences, University of Amsterdam, Amsterdam, The Netherlands

The ongoing SARS-CoV‑2 pandemic and the associated lung disease COVID-19 have led to unprecedented apprehension worldwide. This has resulted in equally unprecedented preventive measures mandated by national governments as well as to an explosion of scientific activity in the search for, among many aspects of the disease, preventive therapy. Central to this search for preventive therapy is the repurposing of chloroquine (CQ) and hydroxychloroquine (HCQ), a traditional antimalarial drug (CQ) and a drug (HCQ) also used in the treatment of systemic lupus erythematosus (SLE) and rheumatoid arthritis (RA). These drugs have surfaced as a potential effective treatment option based on reasonable experimental data but poorly conducted (first) clinical trials. Despite an unconfirmed efficacy and potential serious side-effects the presidents of France, Brazil and the United States subsequently publicly promoted the use of HCQ, resulting in an absolute run on HCQ, a shortage of the drug for patients with traditional indications, and a lively debate in the respective countries [1, 2].

Cardiac side-effects of these drugs have been known for years but have been considered mild. One potentially lethal side-effect is QTc prolongation, caused by blocking of the human ether-a-go-go-related gene (hERG) potassium channel, which results in a prolonged action potential duration [3]. Due to the potentially lethal nature of this side-effect, electrocardiogram (ECG) monitoring in patients treated with these drugs for malaria, SLE or RA has been proposed in the past, but is currently not standard practice [4]. Actually, CQ is estimated to be one of the drugs to which human beings are exposed most [5]. Yet, CQ and HCQ are listed on the website www.crediblemeds.org in the category ‘known risk of causing torsades de pointes’. However, these cases are rare and mostly associated with an (intentional) overdose or in combination with other QTc-prolonging factors (e.g. other drugs, hypokalaemia) and, therefore, CQ and HCQ are generally considered safe with only relatively minor QTc prolongation [5, 6]. Yet, a recent survey of new user cohort studies (2000–2020) in almost a million HCQ users revealed slight excess cardiovascular mortality most likely due to sudden death [7].

Although initial reports, on studies with serious methodological flaws and small sample sizes, seemed to show a beneficial effect on the disease course [8–10], evidence is accumulating that in hospitalised patients HCQ is not effective in reducing mortality [11, 12] or faster virus elimination [13]. In fact, one retrospective study comprising 368 patients found an increased risk of mortality for HCQ, although this could be the result of baseline dissimilarities between the intervention and the control group [12]. This negative effect does not necessarily mean that CQ or HCQ are useless in the setting of a SARS-CoV‑2 infection. It is quite conceivable, based on their demonstrated in vitro efficacy, that treatment in earlier stages of infection might be beneficial. This reasoning underlies the many randomised trials that have been proposed and initiated, also focusing on prevention of infection (i.e. pre-exposure), mainly in healthcare workers, and post-exposure shortly after the onset of disease symptoms [14].

In two studies in this issue of this journal, the effect of CQ was studied in hospitalised patients with emphasis on the main side-effect of the drug, i.e. prolongation of the QTc interval. Outcome data were not included in these reports. In the study by van den Broek et al., 95 patients were treated with CQ (loading dose 600 mg, followed by 2 × 300 mg for 4 days; 22% of patients in the intensive care unit), which resulted in a mean QTc prolongation of 35 ms (95% confidence interval 28–43 ms) [15]. In 22 patients (23%) the QTc interval exceeded 500 ms, which is generally regarded as the value at which to stop all QT-prolonging medication. Interestingly, in addition to QTc prolongation, the authors also detected PR and QRS prolongation, a known effect of CQ [5]. This indicates a decrease in conduction, which is also potentially pro-arrhythmic by favouring re-entrant circuits. In the study by Sinkeler et al. 397 hospitalised patients were treated with CQ (same dosage regimen) [16]. After 24–72 h the QTc had increased by 20 ms (±39 ms, mean ± SD); a second ECG in a subset of patients revealed a further increase to 33 ± 53 ms. In ±16% of patients the QTc interval exceeded 500 ms and/or the increase in QTc was more than 60 ms. One patient developed a non-sustained ventricular tachycardia. Although in both studies serum potassium levels were measured and the use of additional QTc-prolonging drugs was recorded, details are not given in relation to the QTc prolongation. Also, in both studies the computer-measured ECG overestimated the QTc interval, so in patients where clinical decision-making depends on the QTc interval a manual measurement is mandatory.

These results are in line with other published data summarised in a recent review [17]. Up to 20% of patients develop QTc prolongation into the range ≥500 ms with CQ-HCQ monotherapy and slightly more when azithromycin is added [17]. However, only rarely does a patient develop torsades de pointes; a nice example is described by Szekely et al. [18]. Specific subgroups at high risk are recognised and should undergo extra-intense ECG monitoring [19].

An important finding of both studies and other studies on this topic is that COVID-19 patients are apparently much more sensitive to CQ- or HCQ-related QTc prolongation than patients with more conventional indications. This is potentially explained by a number of factors which may contribute to the QT prolongation. In hospitalised COVID-19 patients there is an exaggerated immune response, resulting in high levels of cytokines, including interleukin 6, which has been shown to prolong repolarisation [20]. Furthermore, hypoxia may augment the late sodium inward current, and subsequently prolong the action potential duration [21]. In addition, sick patients may use other QT-prolonging drugs, including the above-mentioned drug azithromycin, and may present with hypokalaemia. Finally, genetic factors particularly present in black African individuals may predispose them to accumulation of all these effects [22]. The hypothesis that QT-prolonging factors, as mentioned above, are imperative in COVID-19 patients is underscored by the fact that the baseline mean QTc interval in COVID-19 patients as presented in both Dutch studies [15, 16] is much longer (in the range 440–450 ms) than in studies with volunteers (400–410 ms) [6].

How should we proceed? Clearly it is a bad idea to provide every citizen with either CQ or HCQ before more information on their efficacy is known. Instead, large-scale, randomised, double-blinded trials are the highest priority in order to prove the efficacy of either drug [1]. These trials should focus on mildly affected patients early after disease onset in order to demonstrate whether these drugs can cure an early infection with SARS-CoV‑2. Secondly, similar studies should be performed in individuals with a high level of exposure (e.g. healthcare workers) to demonstrate whether these drugs are effective in preventing an infection with SARS-CoV‑2. In both groups careful ECG monitoring is warranted to prevent excessive QTc prolongation and, with longer treatment in the pre-exposure group, conduction disturbances. For these prophylactic studies the same motto applies as for the aforementioned presidents: ‘first, do no harm’ [23].
